# T Cell-Association of Carboxy-Terminal Dendrimers with Different Bound Numbers of Phenylalanine and Their Application to Drug Delivery

**DOI:** 10.3390/pharmaceutics15030888

**Published:** 2023-03-09

**Authors:** Hiroya Shiba, Tomoka Hirose, Yunshen Fu, Masataka Michigami, Ikuo Fujii, Ikuhiko Nakase, Akikazu Matsumoto, Chie Kojima

**Affiliations:** 1 Department of Applied Chemistry, Graduate School of Engineering, Osaka Prefecture University, 1-1, Gakuen-cho, Naka-ku, Sakai 599-8531, Osaka, Japan; 2 Department of Biological Science, Graduate School of Science, Osaka Prefecture University, 1-1, Gakuen-cho, Naka-ku, Sakai 599-8531, Osaka, Japan

**Keywords:** dendrimer, drug delivery system, immune cells, phenylalanine, T cells

## Abstract

T cells play important roles in various immune reactions, and their activation is necessary for cancer immunotherapy. Previously, we showed that polyamidoamine (PAMAM) dendrimers modified with 1,2-cyclohexanedicarboxylic acid (CHex) and phenylalanine (Phe) underwent effective uptake by various immune cells, including T cells and their subsets. In this study, we synthesized various carboxy-terminal dendrimers modified with different bound numbers of Phe and investigated the association of these dendrimers with T cells to evaluate the influence of terminal Phe density. Carboxy-terminal dendrimers conjugating Phe at more than half of the termini exhibited a higher association with T cells and other immune cells. The carboxy-terminal Phe-modified dendrimers at 75% Phe density tended to exhibit the highest association with T cells and other immune cells, which was related to their association with liposomes. A model drug, protoporphyrin IX (PpIX), was encapsulated into carboxy-terminal Phe-modified dendrimers, which were then used for drug delivery into T cells. Our results suggest the carboxy-terminal Phe-modified dendrimers are useful for delivery into T cells.

## 1. Introduction

The development of nanocarriers has led to significant advances in the fields of nanotechnology and biotechnology. Nanocarriers are able to deliver bioactive compounds into target cells as well as to control their biodistribution. They are useful for enhancing pharmacological effects and reducing side effects in drug delivery systems (DDS) [1,2]. There are various types of drug nanocarriers such as polymeric micelles, liposomes, silica nanoparticles, and dendrimers [3,4]. Dendrimers are macromolecules with highly branched and well-defined structures that are synthesized via stepwise reactions. Thus, it is easy to control the molecular weight, particle size, and surface structure [5,6]. Moreover, dendrimers can incorporate bioactive compounds, such as drugs, peptides, and ligands by encapsulating or modifying them within or on their surfaces. Therefore, many researchers have studied the applications of dendrimers as potent nanocarriers in DDS. For example, anticancer drugs have been loaded onto poly(ethylene glycol)-modified dendrimers. Ligand peptide-conjugated dendrimers have also been studied for targeting tumor cells and other cells [5,6]. The design of the surface properties of DDS nanocarriers is important. It has been reported that the multivalent binding ability of nanocarriers is enhanced by controlling the density and mobility of ligand molecules on the nanoparticles [7,8]. Phenylketonuria (PKU) is a disorder of phenylalanine (Phe) metabolism. Phe exhibits self-assembling properties in patients with PKU [9,10]. Fibril structures are formed at high Phe concentrations, which enhance protein aggregation, interactions with cell membranes, and membrane permeability [11,12,13,14,15]. These phenomena encouraged the use of Phe-modified nanoparticles as potential drug carriers. Moreover, the Phe density at the surface of drug carriers may affect their cell association properties.

Cancer immunotherapy is one of the cancer treatments, that attacks tumor cells by using the patient’s own immune system. It has gained much attention since the success of immune checkpoint inhibitors in the clinic [16,17]. Although there are various kinds of immune cells, T cells play an important role in cancer immunotherapy. Because it is difficult to deliver into T cells directly, the activities of T cells are controlled by activating macrophages and dendritic cells at present [18,19,20]. Although antibody-conjugated poly (lactic-co-glycolic acid) nanoparticles (NPs) were previously developed for delivery to T cells [21,22], the construction of efficient delivery systems inside T cells is desirable.

**Figure 1:**
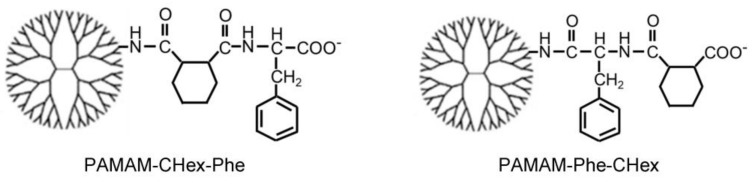
Structure of CHex- and Phe-modified dendrimers.

Because the immune cells in lymph nodes surrounding tumor tissues are important for cancer immunotherapy [23,24], delivery into lymph node-resident immune cells, especially T cells, is useful. It was reported that small nanoparticles less than 70 kDa can be distributed in lymph nodes to access the T cell and B cell zones [25]. It seems that polyamidoamine (PAMAM) dendrimers of less than generation 6 (G6), whose molecular weight is less than 58 kDa, are useful for delivery into lymph node-resident T cells. In our previous studies, the anionic terminal PAMAM dendrimers of greater than G4 were efficiently accumulated in lymph nodes after intradermal injection, but cationic and nonionic terminal dendrimers were not [26]. Moreover, the terminal structure of anionic dendrimers largely affected the cell association properties [27]. Inspired by PKU, carboxy-terminal PAMAM dendrimers of G4 bearing both 1,2-cyclohexanedicarboxylic acid (CHex) and phenylalanine (Phe) such as PAMAM-CHex-Phe and PAMAM-Phe-CHex were synthesized as a nanocarrier for delivery into lymphatic cells (Figure 1). These dendrimers were highly associated with immune cells, including T cells (CD3+ T lymphocytes and splenocytes and Jurkat cells) and their subsets [28,29]. Their cellular uptake was enhanced under weakly acidic conditions (pH 6.5) observed in the tumor microenvironment. Moreover, confocal imaging indicated that these dendrimers were internalized into T cells [29].

We investigated the effect of Phe density at the dendrimer surface on the association with T cells. The surface density of the dendrimer can be controlled by the bound number at the termini and/or dendrimer generation. Thus, dendrimers are useful for performing comprehensive studies to elucidate the relationship between structure and function. Previously, carboxy-terminal dendrimers fully modified with Phe on their terminal groups were examined [29]. In this study, carboxy-terminal dendrimers modified with different bound numbers of Phe were synthesized, and their association with immune cells, especially T cells, was examined. Besides, a model drug, protoporphyrin IX (PpIX), was encapsulated into CHex- and Phe-modified dendrimers to demonstrate the drug delivery ability of the dendrimers into T cells.

## 2. Materials and Methods

### 2.1. Synthesis

In this study, PAMAM-CHex-Phe and PAMAM-Phe-CHex dendrimers with different bound numbers of Phe were synthesized (Table 1). Fully modified PAMAM-CHex-Phe was synthesized, as previously described [29,30]. Briefly, 56 mg of amino-terminal ethylenediamine core PAMAM dendrimer of G4 (Sigma-Aldrich Co., St. Louis, MO, USA) was dissolved in 125 mM NaHCO_3_ aqueous solution (5.5 mL), and approximately 100 equivalents of *cis*-1,2-cyclohexanedicarboxylic anhydride (Tokyo Chemical Industry Co., Ltd., Tokyo, Japan) was added to the dendrimer solution. The pH of the dendrimer solution was adjusted to approximately 10 using 4 M aqueous NaOH. After stirring overnight at room temperature, the dendrimer was purified by dialysis (MWCO 2 k) in distilled water (1 L, three times), and PAMAM-CHex was obtained from an aqueous solution after lyophilization using a freeze dryer (FDM-1000, EYELA, Tokyo, Japan). Then, PAMAM-CHex (53 mg) was dissolved in 5 mL of dimethyl sulfoxide (DMSO anhydrous, Sigma-Aldrich Co.), and L-phenylalanine benzyl ester 4-toluenesulfonate salt (Phe-OBzl·Tos, Peptide Institute, Inc., Osaka, Japan, 0.12 g), 2-(1H-benzotriazole-1-yl)-1,1,3,3-tetrametyluronium hexafluorophosphate (HBTU, Watanabe Chemical Industries, Ltd., Hiroshima, Japan, 0.09 g), and triethylamine (TEA, Nacalai Tesque, Inc., Kyoto, Japan, 41 μL) were added to the dendrimer solution. After stirring for 4 days at room temperature, the dendrimer was purified by dialysis in methanol (1 L, three times) and lyophilized to obtain PAMAM-CHex-Phe-OBzl. Then, PAMAM-CHex-Phe-OBzl (84 mg) was dissolved in methanol (4 mL), and 4 M NaOH methanol solution (0.5 mL) was added. After stirring for 2 h on ice, the dendrimer was dialyzed in distilled water (1 L, three times), and PAMAM-CHex-Phe was obtained after the lyophilization. PAMAM-CHex-Phe dendrimers modified with different bound numbers of Phe were synthesized as described above, except for the reaction ratios of Phe to PAMAM-CHex. The reaction ratios were shown in the Supplementary Materials (Table S1).

In this study, we used PAMAM-Phe-CHex dendrimers bearing different bound numbers of Phe synthesized in our previous reports [29,31].

Fluorescein isothiocyanate (FITC, Tokyo Chemical Industry Co., Ltd.) was conjugated into these dendrimers; 5–10 mg of PAMAM-CHex-Phe and PAMAM-Phe-CHex dendrimers were dissolved in 100 mM of NaHCO_3_ aqueous solution (0.5 mL), and 15 equivalents of N-tert-butoxycarbonyl)-1,2-diaminoethane (Boc-ethylenediamine, Tokyo Chemical Industry Co., Ltd.), and 6 equivalents of 4-(4,6-dimethoxy-1,3,5-triazin-2-yl)-4-methylmorpholinium chloride (DMT-MM, Fujifilm Wako Pure Chemical Co., Tokyo, Japan) were added to the dendrimer solutions. After stirring overnight at room temperature, the dendrimers were purified by Amicon^®^Ultra (MWCO 3 kDa, Merck Millipore, Darmstadt, Germany) using NaHCO_3_ aqueous solution (125 mM), and Boc-ethylenediamine-modified dendrimers were obtained after lyophilization. Each Boc-ethylenediamine-modified dendrimer was stirred in TFA (1 mL) for 3 h on ice. After lyophilization, partially amino-terminal PAMAM-CHex-Phe and PAMAM-Phe-CHex dendrimers were obtained. These dendrimers were dissolved in DMSO (1 mL). Six equivalents of FITC and an excess of TEA were added to the dendrimer solution and then stirred for two days at room temperature. After diluting with water until the DMSO content was less than 2.5%, the reaction mixtures were purified by ultrafiltration (MWCO 3 kDa). FITC-labeled dendrimers were obtained after lyophilization.

### 2.2. Characterization

Proton nuclear magnetic resonance (^1^H-NMR) spectra were recorded using a 400 MHz instrument (JEOL Ltd., Tokyo, Japan) to estimate the bound number of Phe and CHex to the dendrimer. The UV-Vis spectra of the FITC-labeled dendrimers were measured by using Jasco Model V630 UV/Vis spectrophotometer (JASCO Inc., Tokyo, Japan). The bound number of FITC was estimated from the calibration curve and the absorbance at 513 nm in the spectra.

### 2.3. Association of Dendrimers with Immune Cells

Various PAMAM-CHex-Phe and PAMAM-Phe-CHex dendrimers were associated with spleen-derived immune cells using fluorescence-activated cell sorting (FACS), as described in our previous report [29]. Briefly, spleens were collected from BALB/c mice (7–8-week-old, female) after perfusion with phosphate-buffered saline (PBS, pH 7.4) containing heparin under anesthesia by flowing isoflurane at a rate of 0.2 L/min until the mice stopped moving. Splenocytes were obtained after digestion with collagenase for 30 min at 37 °C, and then 2 × 10^5^ cells were suspended in serum-free RPMI-1640 and incubated with each FITC-conjugated PAMAM-CHex-Phe and PAMAM-Phe-CHex dendrimer for 3 h at 37 °C with FITC concentration of 5 μM. After washing with a staining buffer (1% FBS and 2 mM ethylenediaminetetraacetic acid (EDTA) in PBS) once, the cells were stained with phycoerythrin (PE)-conjugated monoclonal antibodies (CD3-PE, CD45R-PE, F4/80-PE, and CD11c-PE) purchased from Miltenyi Biotec GmbH, Ltd., Bergish Gladbach, Germany, according to the manufacturer’s instructions. The stained and non-stained cells were analyzed using a BD FACSAria™ III Cell Sorter (Becton, Dickinson and Company, Franklin Lakes, NJ, USA), and the mean green fluorescence intensity of cells stained with each PE-labeled antibody was measured. All animal experiments were performed according to protocols approved by the Animal Care and Use Committees of Osaka Metropolitan University (No. 22-22) and Osaka Prefecture University (No. 21-100).

Association of various PAMAM-CHex-Phe and PAMAM-Phe-CHex dendrimers with Jurkat cells was also performed using GUAVA^®^ InCyte™ (Luminex, Japan), in accordance with our previous report [29]. Briefly, RPMI solutions containing each FITC-conjugated PAMAM-CHex-Phe and PAMAM-Phe-CHex dendrimer (FITC 5 μM) were added to 1 × 10^5^ Jurkat cells and incubated for 3 h at 37 °C. Then, PBS was added to the cell suspension and centrifuged to collect the cells. After washing with 400 μL PBS once, FACS was performed to measure the mean green fluorescence intensity. The same procedure was performed in the incubation of PAMAM-CHex-Phe and PAMAM-Phe-CHex dendrimers at pH 6.5.

### 2.4. Adsorption of Dendrimers to Liposomes

The adsorption of PAMAM-CHex-Phe and PAMAM-Phe-CHex dendrimers with different bound numbers of Phe onto liposomes was performed in accordance with our previous report [29]. Briefly, a chloroform solution of hydrogenated soy phosphatidylcholine (HSPC, NOF Corp., Tokyo, Japan; 10 mg/mL) was dried overnight under vacuum, then dissolved in 0.1 M 4-(2-hydroxyethyl)-1-piperazineethanesulfonic acid (HEPES) buffer (pH 7.4) and sonicated for 2 min using a bath sonicator (ASU-6, AS ONE Corp., Osaka, Japan) to obtain dispersed liposomes (HSPC: 1.6 mg/mL). 25 μL of the FITC-conjugated PAMAM-CHex-Phe and PAMAM-Phe-CHex dendrimer aqueous solutions (FITC: 100 μM) were mixed with 475 μL of the liposome solution and incubated for 3 h at 37 °C. After the liposomes were precipitated by centrifugation (37 °C, 11,000 rpm, 15 min) and subsequently washed with HEPES buffer, chloroform/methanol (1/1, 0.5 mL) was added to the precipitate and shaken for 20 min at 37 °C, in order to dissolve the liposome. The fluorescence intensity (F) was measured using an FP-6200 spectrofluorometer (JASCO Inc.). The excitation and emission wavelengths were 495 nm and 520 nm, respectively. The adsorption ratio of dendrimers to liposomes was calculated using the following equation:

Adsorption ratio (%) = F/F_den_ (1)

where F_den_ is the fluorescence intensity of each FITC-conjugated PAMAM-CHex-Phe and PAMAM-Phe-CHex dendrimer solution (FITC: 5 μM).

### 2.5. Encapsulation of PpIX in Dendrimers

PpIX was purchased from Sigma-Aldrich Co. PAMAM-CHex-Phe and PAMAM-Phe-CHex dendrimer solutions (0.1 mM) and 20 mM PpIX solution were prepared using DMF. Ten equivalents of PpIX were added to each dendrimer, and the mixture was stirred at 40 °C for 30 min. The solution was then evaporated and dried overnight under a vacuum. Deionized water (200 μL) was added to each sample to prepare 0.1 mM dendrimer aqueous solutions. The solutions were then centrifuged (14,000 rpm, 25 °C, 20 min). DMF (990 μL) was added to 10 μL of the supernatant, the UV-Vis spectra were measured using a Jasco Model V630 UV/Vis spectrophotometer (JASCO Inc.). The amount of encapsulated PpIX was calculated by measuring the absorbance at a wavelength of 406 nm, based on the calibration curve.

### 2.6. Delivery of PpIX-Encapsulating Dendrimers into T Cells

Splenocytes were obtained, as described in Section 2.3. 2 × 10^5^ Cells were suspended in serum-free RPMI-1640 medium and incubated with PpIX-loaded dendrimer solutions (PAMAM-CHex-Phe64 and PAMAM-Phe64-CHex) for 3 h at 37 °C at a PpIX concentration of 5 μM. After washing with the staining buffer once, the cells were stained with CD3-PE-conjugated monoclonal antibody and analyzed by FACS, as described in Section 2.3. PpIX and PE were excited by 633 nm and 561 nm lasers, respectively, and the mean PpIX fluorescence intensity of cells stained with CD3-PE-labeled antibodies was analyzed.

## 3. Results and Discussion

### 3.1. Synthesis of Carboxy-Terminal Dendrimers Modified with Different Bound Numbers of Phe

**Figure 2:**
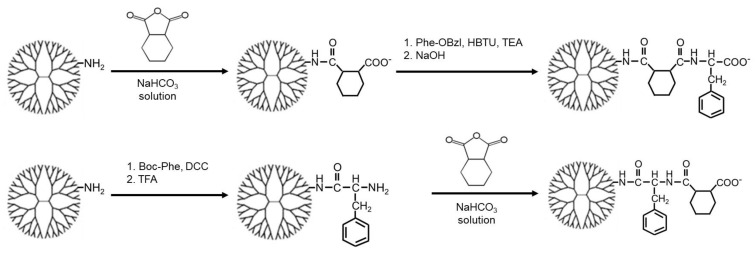
Synthetic schemes of PAMAM-CHex-Phe (**top**) and PAMAM-Phe-CHex (**bottom**).

In this study, CHex- and Phe-modified dendrimers with different bound numbers of Phe were used to investigate the influence of the dendrimer terminal Phe density on cell association and drug delivery ability. Two types of CHex- and Phe-modified dendrimers, PAMAM-CHex-Phe and PAMAM-Phe-CHex, were synthesized by varying the reaction order of *cis*-1,2-cyclohexanedicarboxylic anhydride and Phe, as shown in Figure 2 [30,31]. In the synthesis of PAMAM-CHex-Phe, the PAMAM G4 dendrimer was reacted with an excess of *cis*-1,2-cyclohexanedicarboxylic anhydride, followed by a reaction with Phe-OBzl·Tos and the subsequent deprotection. In the synthesis of PAMAM-Phe-CHex, the PAMAM dendrimer was first reacted with Boc-Phe. After deprotection, an excess of *cis*-1,2-cyclohexanedicarboxylic anhydride was reacted. The bound numbers of Phe in these dendrimers were regulated by varying the Phe/PAMAM ratio in the reaction. The average bound numbers of Phe were evaluated from the integral rations of the signals at 7.0–7.4 ppm and 4.2 ppm (Phe) to 2.2 ppm (PAMAM) in the ^1^H NMR spectra of PAMAM-CHex-Phe and PAMAM-Phe-CHex dendrimers. The bound number of CHex was evaluated from the integral ratios of the signals at 1.1–1.9 ppm (CHex) and 7.0–7.4 (Phe) to 2.2 ppm (dendrimer) in the ^1^H NMR spectra (see Supplementary Materials). Then, these dendrimers were labeled with FITC after adding small amounts of amino groups to the dendrimer, as described in our previous report [28,29]. The dendrimers used in this study are listed in Table 1. Three to seven FITC molecules were conjugated to each dendrimer. These FITC-label dendrimers were used for the analysis of cell association.

### 3.2. Cell Association of Carboxy-Terminal Dendrimers Modified with Various Numbers of Phe

**Figure 3:**
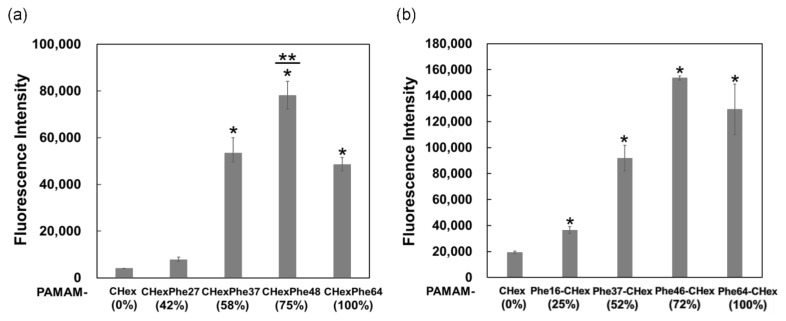
T cell-association of PAMAM-CHex-Phe (**a**) and PAMAM-Phe-CHex (**b**) dendrimers. * *p* < 0.05 vs. PAMAM-CHex ** Higher and *p* < 0.05 vs. PAMAM-CHex-Phe64 or PAMAM- Phe64-CHex.

The T cell association of PAMAM-CHex-Phe and PAMAM-Phe-CHex dendrimers with different bound numbers of Phe was examined by using CD3-positive splenocytes as a model of T cells. Splenocytes were incubated with FITC-labeled PAMAM-CHex-Phe and PAMAM-Phe-CHex dendrimers and then stained with CD3-PE. Double-positive cells indicate dendrimer-associated T cells. The mean fluorescence intensity of CD3-positive cells was measured by FACS. In Figure 3, the fluorescence intensity of PAMAM-CHex-Phe and PAMAM-Phe-CHex was much higher than that of PAMAM-CHex without Phe, indicating that PAMAM-CHex-Phe and PAMAM-Phe-CHex were associated much more efficiently with T cells than PAMAM-CHex without Phe. These results were consistent with our previous studies [28,29]. These cell association properties were largely dependent on the bound number of Phe, as expected. The association with T cells increased significantly when the bound number of Phe was greater than 37 (58% of the terminal groups of dendrimers) in PAMAM-CHex-Phe dendrimers. PAMAM-CHex-Phe48 exhibited the highest degree of cell association. The association of PAMAM-Phe-CHex dendrimers with T cells was similar to that of the PAMAM-CHex-Phe dendrimers. These results suggest that CHex- and Phe-modified dendrimers at more than 50% Phe density are useful for the T cell association. It has also been suggested that T cell association can be improved by optimizing Phe density at the dendrimers.

**Figure 4:**
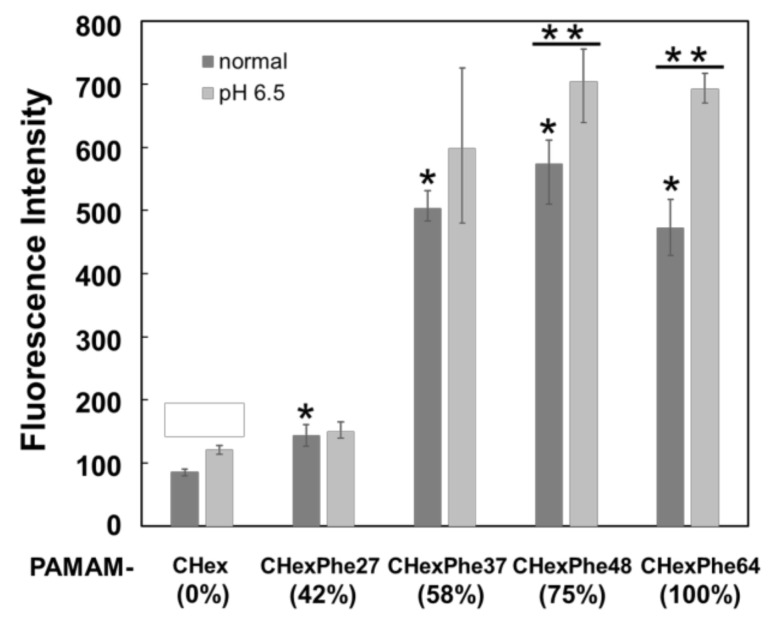
Association of PAMAM-CHex-Phe with Jurkat cells. * *p* < 0.05 vs. PAMAM-CHex. ** *p* < 0.05 vs. normal condition.

PAMAM-CHex-Phe64 and PAMAM-Phe64-CHex have been shown to exhibit pH-dependent T cell-association properties because these dendrimers became turbid at weakly acidic pHs due to the protonation of terminal carboxyl groups [29,30]. It was reported that high concentrations of lactate, a product of glycolysis by tumor cells, inhibit effector T cell proliferation and cytokine production [32]. Thus, for efficient cancer immunotherapy, delivery into T cells in weakly acidic conditions is important. The pH dependency in cell association of dendrimers with different bound numbers of Phe was examined using Jurkat cells as another model of T cells. Figure 4 shows that PAMAM-CHex-Phe was associated much more efficiently with T cells than PAMAM-CHex without Phe. The association with T cells increased significantly, when the bound number of Phe was greater than 37 (58% of the terminal groups of dendrimers). The highest cell association was observed for the dendrimers with 75% Phe density. This cell association property of these dendrimers to Jurkat cells was similar to that to spleen-derived T cells. In addition, the association of PAMAM-CHex-Phe48 and PAMAM-CHex-Phe64 with Jurkat cells increased at pH 6.5 compared to the association under normal conditions. However, the pH sensitivity tended to decrease with decreasing bound numbers of Phe. Our previous report indicated that the stimuli sensitivity broadened with decreasing bound numbers of Phe in the dendrimer [31], which is consistent with this result. Thus, our results indicate that the bound number of Phe in the dendrimer is involved not only in the T cell-association property but also in the expression of pH responsiveness.

**Figure 5:**
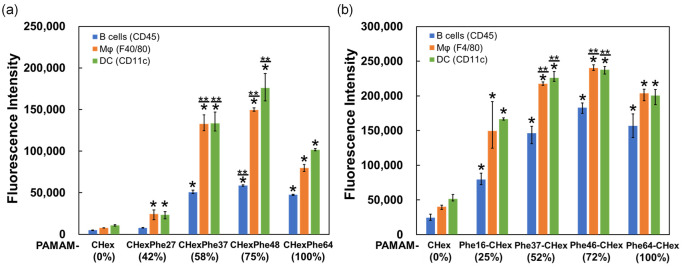
Association of PAMAM-CHex-Phe (**a**) and PAMAM-Phe-CHex (**b**) dendrimers with various immune cells. * *p* < 0.05 vs. PAMAM-CHex ** Higher and *p* < 0.05 vs. PAMAM-CHex-Phe64 or PAMAM-Phe64-CHex.

PAMAM-CHex-Phe and PAMAM-Phe-CHex dendrimers, with Phe densities greater than 50%, were highly associated with T cells and the highest cell association was observed for the dendrimers with 75% Phe density. To examine the association of these dendrimers with other immune cells, their association with B cells, macrophages (Mϕ), and dendritic cells (DC) was examined by staining with CD45R-PE, F4/80-PE, and CD11c-PE, respectively. A similar cell association trend for PAMAM-CHex-Phe and PAMAM-Phe-CHex dendrimers was observed in these immune cells. CHex- and Phe-modified dendrimers with more than 50% Phe density exhibited higher cell association. Moreover, it is likely that the highest cell association was observed for the dendrimers with around 75% Phe density (Figure 5). These results suggest that Phe modification at more than half of the termini of the PAMAM G4 dendrimer is necessary to enhance the association with immune cells and cell association can be improved by optimizing Phe density at the dendrimers.

**Figure 6:**
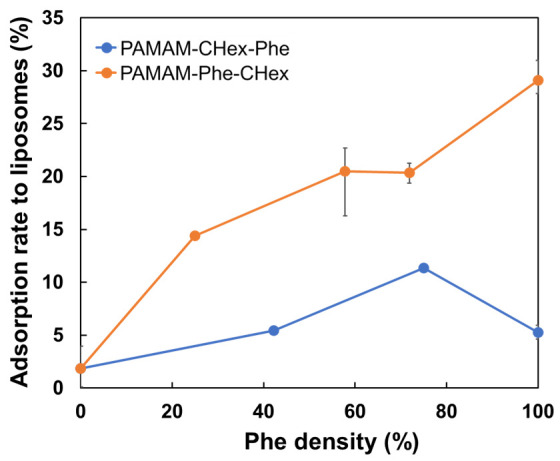
Adsorption of PAMAM-CHex-Phe and PAMAM-Phe-CHex dendrimers with different Phe densities to liposomes.

Similar results were obtained in our cell association experiments using different types of cells. CHex-and Phe-modified dendrimers at more than 50% Phe density, especially 75% Phe density, enhanced the association with various cells. This suggests that the physical properties of CHex- and Phe-modified dendrimers are largely involved in improving the cell association of dendrimers. The interactions between dendrimers and the cell membrane were investigated using liposomes as a model of the cell membrane. Figure 6 shows that the adsorption ratio of PAMAM-Phe-CHex to the liposomes increased, as the bound number of Phe increased. The adsorption ratio reached a maximum at a Phe density of 75% in the PAMAM-CHex-Phe dendrimers. These results are consistent with our cell association results. The hydrophobicity of dendrimers is an important factor for cell association. The addition of Phe to the dendrimer increased the Log *P* (octanol/water partition coefficient) value, which increased with increasing Phe bound numbers [28,33]. Previously, Shima et al. studied poly(γ-glutamic acid) (PGA) NPs grafting various bound numbers of Phe, indicating that higher cell association of Phe-grafted PGA NPs was observed in PGA NPs with higher Phe grafting ratios [34]. This report supports our results. However, the highest cell association was observed for the dendrimers with 75% Phe density, but not for those with 100% Phe density. This phenomenon was not observed in Phe-grafted PGA NPs [34], because the flexibility and density of Phe at the surface of PGA NPs might be different from those of our dendrimers. Spherical NPs with high hydrophobicity and rigid structures tend to show higher immune cell uptake than worm-like NPs with low hydrophobicity and rigidity [35]. Thus, cell association was affected not only by hydrophobicity but also by Phe density. The self-assembled structures of Phe enhance the association with the cell membrane and membrane permeability, which are observed in patients with PKU [11,12,13,14,15]. Griffith et al. have reported that aggregated Phe structures enhance cell membrane permeability. An aggregated structure is formed at the lipid membrane interface [13]. Moreover, hydrophobic aromatic side chains are exposed outside the aggregated structure [12]. Thus, the Phe conformation at the nanoparticle surface is also important for cellular uptake. The diameter of the PAMAM G4 dendrimer is estimated as ~4.5 nm [3], and the cross-sections of Phe termini in assumed spherical dendrimers at 100%, 75%, and 50% Phe densities were calculated as 0.95, 1.3, and 1.9 nm^2^, respectively. The cross-section of the Phe monomer in the assembled structure was estimated to be 0.73 nm^2^ [23], which is comparable to the Phe density at the dendrimer termini. This supported our findings that Phe density at the dendrimer termini influenced cell association.

Negatively charged NPs are preferentially taken up by macrophages and DCs, which have scavenger receptors [36]. L-type amino acid transporter 1 (LAT1) is an amino acid transporter at the surface of cells, such as T cells, with high proliferative and differentiation potential. Differentiating T cells specifically upregulates LAT1 expression to ensure sufficient nutrients for metabolic reprogramming in response to antigens [37]. LAT1 facilitates the internalization of nanocarriers into tumor cells [38]. Thus, LAT1 is a possible factor contributing towards internalization of PAMAM-CHex-Phe and PAMAM-Phe-CHex into T cells. Further investigation is required to elucidate the cell association mechanism as well as the effect of Phe density on CHex- and Phe-modified dendrimers.

### 3.3. Model Drug Delivery into T Cells Using PAMAM-CHex-Phe and PAMAM-Phe-CHex

**Figure 7:**
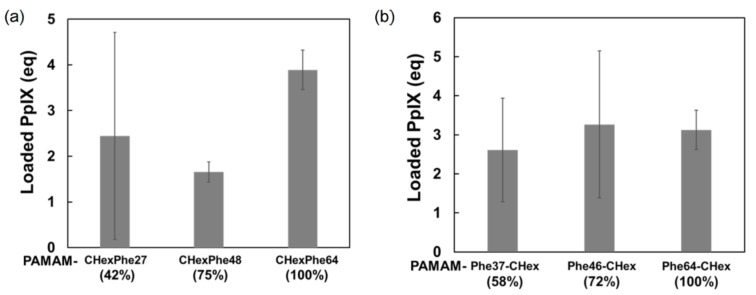
Loading of PpIX in PAMAM-CHex-Phe (**a**) and PAMAM-Phe-CHex (**b**) dendrimer aqueous solutions.

Finally, we attempted to use PAMAM-CHex-Phe and PAMAM-Phe-CHex dendrimers for drug delivery into T cells. Protoporphyrin IX (PpIX) is a water-insoluble photosensitizer that was used as a model drug in this study. The drug-loading ability of the PAMAM-CHex-Phe and PAMAM-Phe-CHex dendrimers was examined, as follows. First, PpIX and each dendrimer were dissolved in DMF, and these solutions were mixed to adjust the mole ratio of PpIX/dendrimer to 10/1. After evaporation and subsequent drying, the residue was dissolved in deionized water. PpIX loaded into the dendrimer was soluble in water, whereas free PpIX was not. Thus, water-soluble PpIX loaded into the dendrimer was collected after centrifugation. The UV-Vis spectrum of each supernatant was measured to estimate the amount of encapsulated PpIX. Both PAMAM-CHex-Phe and PAMAM-Phe-CHex dendrimers encapsulated 2–4 PpIX molecules per dendrimer. As the bound number of Phe in the dendrimer increased, more PpIX molecules tended to be encapsulated (Figure 7). Thus, fully Phe-modified PAMAM-CHex-Phe and PAMAM-Phe-CHex dendrimers were used for PpIX delivery. In our previous study, the encapsulation of guest molecules into PAMAM dendrimers was based on electrostatic and hydrophobic interactions [39]. The electrostatic interactions possibly work between the tertiary amino groups of the PAMAM dendrimer and the carboxyl groups of PpIX. The amount of encapsulated PpIX increased, as the bound number of Phe increased. It may be because the hydrophobic interaction was enhanced between PpIX and Phe.

**Figure 8:**
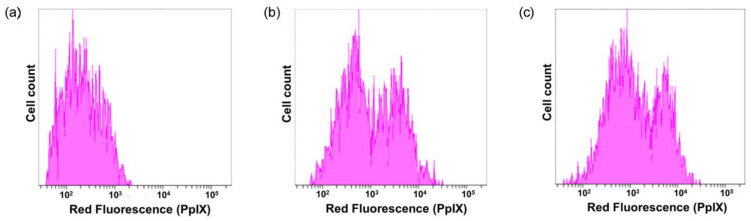
Delivery of PpIX to CD3-positive T cells. Cells only (**a**), PpIX-encapsulated PAMAM-CHex-Phe64 (**b**), and PAMAM-Phe64-CHex (**c**).

Next, PpIX-encapsulated dendrimers were added to spleen-derived CD3-positive T cells to examine their ability to deliver PpIX to T cells. Splenocytes were incubated with PpIX-encapsulated dendrimers (PAMAM-CHex-Phe64 and PAMAM-Phe64-CHex) and stained with CD3-PE. The mean PpIX fluorescence intensity of cells stained with CD3-PE-labeled antibodies was analyzed. Figure 8 shows that both PAMAM-CHex-Phe64 and PAMAM-Phe64-CHex enhanced PpIX-derived red fluorescence intensity. These suggest the potential of these dendrimers for drug delivery into T cells. Because the molecular weights of our dendrimers, PAMAM-CHex-Phe and PAMAM-Phe-CHex, are less than 70 kDa, it is expected that these dendrimers are distributed in the T cell and B cell zones of lymph nodes [25]. Our findings possibly contribute to the construction of DDS for delivery into lymph node-resident T cells, although in vivo experiments are required.

## 4. Conclusions

In this study, we synthesized carboxy-terminal dendrimers modified with different bound numbers of Phe to investigate the influence of Phe density at the dendrimer termini on the association with T cells. Both PAMAM-CHex-Phe and PAMAM-Phe-CHex dendrimers at more than 50% Phe density were highly associated with T cells and other immune cells. These dendrimers at 75% Phe density exhibited the highest cell association, indicating the importance of the Phe density of the dendrimers for cell association. These dendrimers encapsulated the model drug PpIX. In our ex vivo experiments, PpIX was delivered into T cells. This suggests that these dendrimers are potent DDS carriers for T cell delivery.

## Figures and Tables

**Table 1:** Dendrimers with different bound numbers of Phe used in this study.

Dendrimer ^1^	Bound Number		
	Phe (Terminal Density ^3^)	CHex	FITC
PAMAM-CHex-Phe64 ^2^	64 (100%)	64	7.0
PAMAM-CHex-Phe48	48 (75%)	64	4.0
PAMAM-CHex-Phe37	37 (58%)	64	3.2
PAMAM-CHex-Phe27	27 (42%)	64	4.1
PAMAM-Phe64-CHex ^2^	64 (100%)	62	4.0
PAMAM-Phe46-CHex ^2^	46 (72%)	~64	4.7
PAMAM-Phe37-CHex ^2^	37 (58%)	~64	3.3
PAMAM-Phe16-CHex ^2^	16 (25%)	~64	2.5
PAMAM-CHex	0 (0%)	64	4.0

^1^ The number of terminal groups of the dendrimer is 64. ^2^ Refers to our previous reports [29,31]. ^3^ Calculated as the percentage of the bound number of Phe to dendrimer terminal number (64).

## Data Availability

Data supporting the findings of this study are available from the corresponding author upon reasonable request.

## Supplementary Materials

The following supporting information can be downloaded at https://www.mdpi.com/article/10.3390/pharmaceutics15030888/s1, Table S1: Reaction ratios for the synthesis of various PAMAM-CHex-Phe dendrimers. Figure S1: Dendrimer structure for the assignment of our NMR spectra. Figures S2–S4: ^1^H NMR spectra of the dendrimers synthesized in this study.
