# Supplementary material for: T Cell-Association of Carboxy-Terminal Dendrimers with Different Bound Numbers of Phenylalanine and Their Application to Drug Delivery
Source: Pharmaceutics. 2023 Mar 9;15(3):888. doi: 10.3390/pharmaceutics15030888 (PMC10052534; doi:10.3390/pharmaceutics15030888)
Supplement: Supplementary file 1 [file pharmaceutics-15-00888-s001.zip › pharmaceutics-2232224-supplementary.pdf]

Supplementary Information

T Cell-Association of Carboxy-Terminal Dendrimers with Different Bound Numbers of Phenylalanine and Their Application to Drug Delivery

Hiroya Shiba [1], Tomoka Hirose [1], Yunshen Fu [1], Masataka Michigami [2], Ikuo Fujii [2], Ikuhiko Nakase [2], Akikazu Matsumoto [1] and Chie Kojima [1,*]

Table S1. Reaction ratios to synthesize various PAMAM-Phe-CHex dendrimers

Dendrimer[1]	Phe	
	in Feed	Bound
PAMAM-CHex-Phe64[2]	142	64
PAMAM-CHex-Phe48	55	48
PAMAM-CHex-Phe37	45	37
PAMAM-CHex-Phe27	37	27

[1] The number of terminal groups of the dendrimer is 64.
[2] Referred to our previous report: Shiba, H. et al. J. Mater. Chem. B. 2022, 10, 2463.

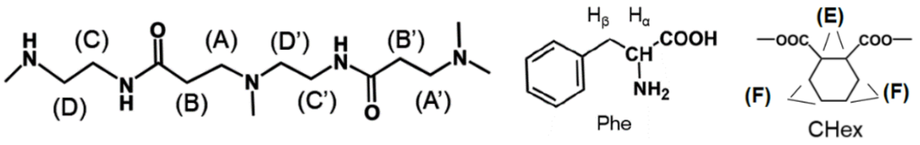

Figure S1 Partial dendrimer structure for assignment for of the following ^{1}H NMR spectra.

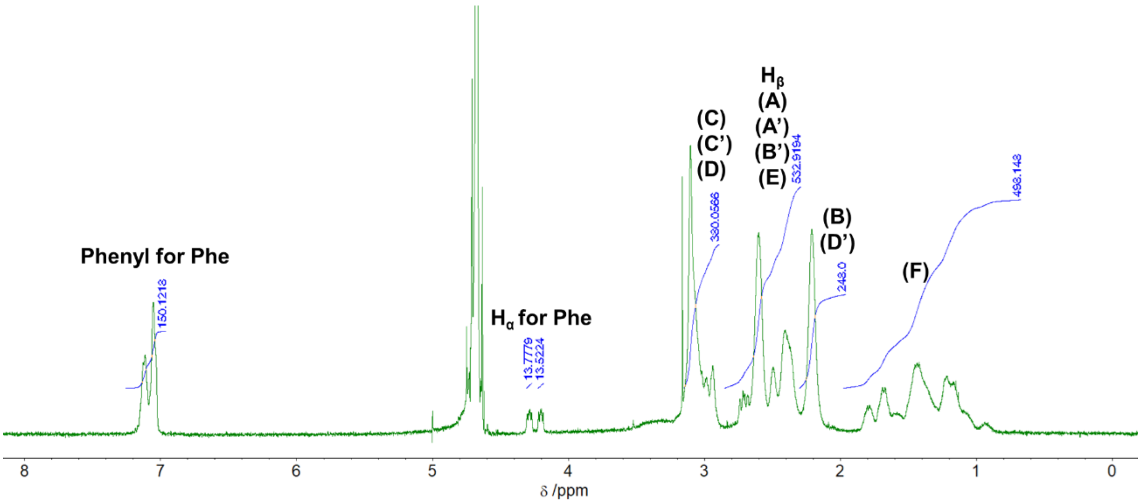

Figure S2. ^{1}H NMR spectrum of PAMAM-CHex-Phe27 in D_2O containing NaOD.

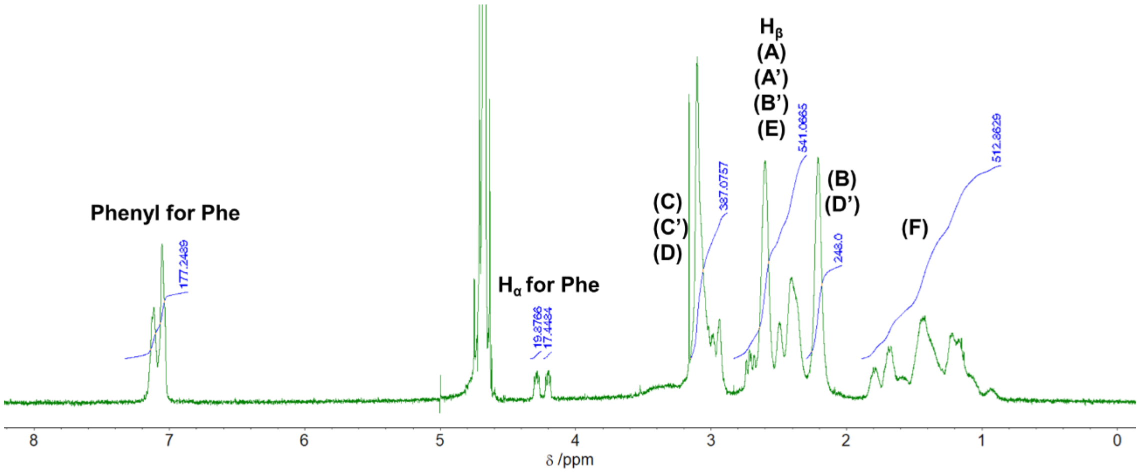

Figure S3. ^{1}H NMR spectrum of PAMAM-CHex-Phe37 in D_2O containing NaOD.

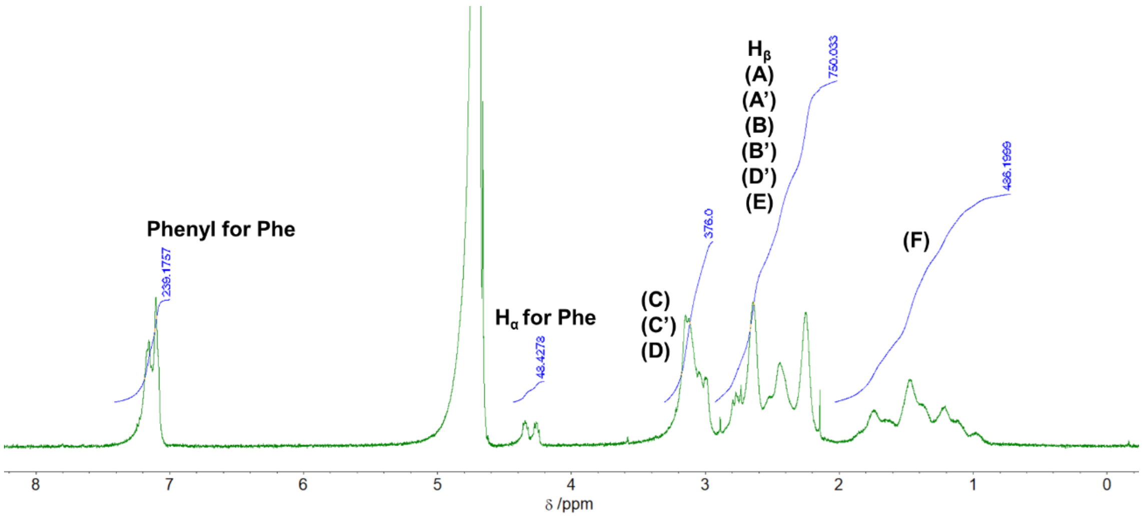

Figure S4. ^{1}H NMR spectrum of PAMAM-CHex-Phe48 in D_2O containing NaOD.
